# The renal microcirculation in chronic kidney disease: novel diagnostic methods and therapeutic perspectives

**DOI:** 10.1186/s13578-021-00606-4

**Published:** 2021-05-17

**Authors:** Shulin Li, Fei Wang, Dong Sun

**Affiliations:** 1 Department of Nephrology, Affiliated Hospital of Xuzhou Medical University, 99 West Huai-hai Road, Xuzhou, 221002 Jiangsu China; 2 Department of Internal Medicine and Diagnostics, Xuzhou Medical University, Xuzhou, 221002 China

**Keywords:** Chronic kidney disease, Microvascular injury, Endothelial dysfunction, Microvascular assessment, Therapeutic strategies

## Abstract

Chronic kidney disease (CKD) affects 8–16% of the population worldwide and is characterized by fibrotic processes. Understanding the cellular and molecular mechanisms underpinning renal fibrosis is critical to the development of new therapeutics. Microvascular injury is considered an important contributor to renal progressive diseases. Vascular endothelium plays a significant role in responding to physical and chemical signals by generating factors that help maintain normal vascular tone, inhibit leukocyte adhesion and platelet aggregation, and suppress smooth muscle cell proliferation. Loss of the rich capillary network results in endothelial dysfunction, hypoxia, and inflammatory and oxidative effects and further leads to the imbalance of pro- and antiangiogenic factors, endothelial cell apoptosis and endothelial-mesenchymal transition. New techniques, including both invasive and noninvasive techniques, offer multiple methods to observe and monitor renal microcirculation and guide targeted therapeutic strategies. A better understanding of the role of endothelium in CKD will help in the development of effective interventions for renal microcirculation improvement. This review focuses on the role of microvascular injury in CKD, the methods to detect microvessels and the novel treatments to ameliorate renal fibrosis.

## Introduction

The number of patients with chronic kidney disease (CKD) and consequently those progressing to end-stage renal disease (ESRD) is increasing, and the social, economic, and medical burden of CKD has received increased attention as a leading public health problem around the world [1]. The prevalence of CKD in China is as high as 10.8% according to a survey published in Lancet in 2012 [2]. Despite traditional drug and renal replacement therapies, the proportion of patients with CKD progressing to ESRD is still high, and the 5-year survival rate of dialysis patients is less than 50%. Therefore, protecting renal function is a leading public health goal, and a better understanding of the mechanisms underlying CKD is urgently needed.

Systemic microvascular rarefaction is one of the pathogenesis of the development of multiorgan damage in CKD patients, for example, in skeletal muscle, heart, and cerebrovascular disease. Flisinki et al. found that the microvascular density was significantly decreased in the locomotor muscle in rats with 5/6 nephrectomy comparing to the sham-operated controls, which may lead to uremic myopathy syndrome [3]. A greater decrease of microvascular rarefaction in the myocardium was also found in a few studies examining the heart in rodents with the CKD models, suggesting a systemic “toxic” effect of uremia on the endothelial cells [4–6]. In a study of Burns et al., 48% of CKD participants had mild or severe cognitive impairment, which may due to the cerebral vessel disease. This implicated that microvascular rarefaction in CKD is one of the pathogenesis of cognitive impairment [7]. Renal capillaries, which deliver oxygen and nutrients to the tubules, mediate vasoactivity, and maintain the local balance between pro- and antiangiogenic factors, are crucial for proper kidney function. Several recent studies have suggested renal capillary rarefaction and endothelial injury as significant pathophysiological processes promoting renal fibrosis [8–12]. Microvascular injury results in increased vasoconstriction, vascular permeability, activation of the complement system, inflammation, oxidative stress, and endothelial cell apoptosis/necrosis, and is thought to be closely linked to the progression of CKD to ESRD. In this review, we analyze the role of microvascular damage in CKD progression, summarize recent developments in experimental technology used to observe tissue microvasculature, and discuss the therapeutic issues related to targeting the restoration of renal capillaries.

## Vascular endothelial function and dysfunction

Blood vasculature is important for the health of tissues as it facilitates the delivery of oxygen and nutrients. Endothelial cell injury is a major event in CKD that contributes to multiple macro- and microvascular damages. Healthy endothelium is considered a key contributor to the response to physical and chemical signals via maintaining barrier function, limiting platelet aggregation and adhesion, maintaining normal vascular tone and vascular homeostasis, regulating anti-inflammatory factors and coordinating immune response. Under physiological conditions, the intact vascular endothelium can effectively prevent pathogen invasion, at the same time, vascular endothelial cells synthesize and release tissue plasminogen activator (tPA), thrombomodulin (TM) and other anticoagulant substances, which make the blood in the blood vessel in a mild fibrinolytic state and maintain smooth blood circulation [13]. When the integrity of the vascular endothelium is destroyed and the collagen under the intima is exposed, platelet adhesion and aggregation will begin, and the aggregated platelets can release ADP, TXA2 and activated platelet activating factor (PAF), which can be triggered and exacerbated platelet aggregation. Endothelial cells and platelets work together to complete the process of hemostasis and blood vessel damage repair [14].

Another important function of endothelium is to regulate vascular tone, which results from the release of several vasoactive molecules that relax the vessel, such as nitric oxide (NO), or constrict the vessel, such as angiotensin-2 and endothelin (ET). NO is a physiological metabolite of L-arginine produced following the activation of endothelial NO synthase (eNOS) [15]. This eNOS-derived NO diffuses to the vascular smooth muscle cells and activates guanylate cyclase, which contributes to cGMP-mediated vasodilatation [16]. NO has diverse biological functions, including relaxing vascular smooth muscle and preventing its proliferation and inhibiting platelet aggregation and leukocyte activation. Therefore, a reduction in vasodilators may result in impaired endothelium-dependent vasorelaxation and subsequently lead to tissue ischemia and hypoxia. Angiotensin-2, the major activator in the renin–angiotensin system (RAS), has been shown to participate in endothelial dysfunction via its vasoconstrictive, proliferative and proinflammatory effects on vessels [17]. A study reported that activation of angiotensin-2 signaling triggers oxidative stress by inducing multiple downstream pathways, thereby resulting in growth arrest and apoptosis in endothelial cells. Blockage of angiotensin-2 signaling has been reported to protect the retinal vasculature by inhibiting endothelial cell apoptosis and improving blood flow [18]. Endothelin (ET) is mainly synthesized by endothelial cells and is a powerful vasoconstrictor. Serum ET-1 level (the main form of endothelin) plays an important role in maintaining basal vascular tone and cardiovascular system homeostasis [19]. Therefore, the imbalance of vasodilatative and vasoconstrictive molecules leads to impaired endothelial function.

The endothelium is also injured in response to the damage of vascular homeostasis, which is causing by an increase in antiangiogenic factors, such as angiopoietin-2 (Ang-2) [20], and thromboxane A2 [21], and a decrease in proangiogenic factors, such as Ang-1 [22, 23], vascular endothelial growth factor (VEGF), and EGF [24]. An imbalance in proangiogenic and antiangiogenic factors results in functional defects and structural abnormalities in microvessels. Ang-1and Ang-2 are natural ligands of the Tie-2 receptor, which is expressed almost exclusively on endothelial cells and hemopoietic cells. Ang-1 could promote endothelial cell stability by binding and inducing tyrosine phosphorylation in Tie-2 [25, 26]. This reaction helps maintain a ‘quiescent’ phenotype in capillaries by inhibiting endothelial hyperpermeability, thereby preventing inflammatory cells from infiltrating renal interstitium and activating fibroblasts. The studies to date have demonstrated that Tie2 participates in angiogenesis, vessel remodeling and vessel maturation. Maisonpierre PC et al. showed that overexpression of Ang-2 could disrupt vessel formation in vivo, indicating that Ang-2 is a naturally competitive antagonist of Ang1 and the Tie2 receptor [27].

As an active part of the immune system, endothelial cells, in addition to ensuring the integrity of the structure and function of the blood vessel wall, can also express or up-regulate a variety of immune function-related molecules after activation, such as IL-1, IL-6, IFNβ, M2CSF, etc. [28]. Endothelial cells can not only serve as target cells for cytokines and inflammatory factors, but also secrete active mediators such as cytokines, and play an important role in the body's inflammatory and immune response processes.

## Microvascular injury and kidney diseases

The kidney is a highly vascularized organ. Microvascular injury has been observed in different kidney diseases, which eventually progress to CKD. Rafael Kramann R et al. [29] directly showed decreased peritubular capillary (PTC) density and diameter of individual capillaries in long-term acute kidney injury (AKI) models by using a novel technique known as fluorescence microangiography (FMA), demonstrating a link between AKI leading to future CKD and loss of peritubular perfusion. Ehling J et al. [30] showed that mice with I/R-induced AKI exhibited significant capillary rarefaction at early time points after I/R injury by using microcomputed tomography (µCT) and 3D microarchitecture to analyze the renal vasculature. Although glomerular hypertrophy and hyperfiltration induced by neoangiogenesis of the microvessels were observed in early diabetes, the opposite was observed in advanced diabetic nephropathy, which is characterized by capillary rarefaction accompanied by local tissue hypoxia [31]. A study by Maric-Bilkan C et al. [32] showed that the density of microvessels with diameters under 100 μm was reduced by 34% after 4 weeks and by 75% after 12 weeks in the streptozotocin-induced diabetic models compared with the nondiabetic models by using micro-CT reconstruction of the renal microvascular architecture. Hypertension is second only to diabetes in leading to the development of CKD [33, 34], which is accompanied by functional and structural alterations in microvessels. Hao HF et al. [35] showed that renal arteries from spontaneous hypertensive rats (SHRs) exhibited less relaxation in response to acetylcholine, decreased phosphorylation of eNOS, and downregulation of NO production, suggesting that endothelial function is impaired in hypertension models. Recently, several studies have demonstrated that hyperuricemia is actively involved in the development of CKD. Kang DH [36] found that uric acid contributed to a phenotypic change in umbilical vein endothelial cells (HUVECs) to elongated fibroblast-like cells accompanied with endothelial cell apoptosis in vitro. Meanwhile, capillary rarefaction and increased fibrosis marked by α-SMA were detected in the animal model of hyperuricemia, demonstrating the progress of fibrosis in the kidney of hyperuricemic rats in vivo. According to the abovementioned studies, ischemia/reperfusion, diabetes, hypertension and hyperuricemia are some of the prevalent risk factors for endothelial dysfunction and injury, which lead to CKD. Moreover, microvascular injury is thought to be the common pathophysiological pathway in the progression to CKD.

**Fig. 1:**
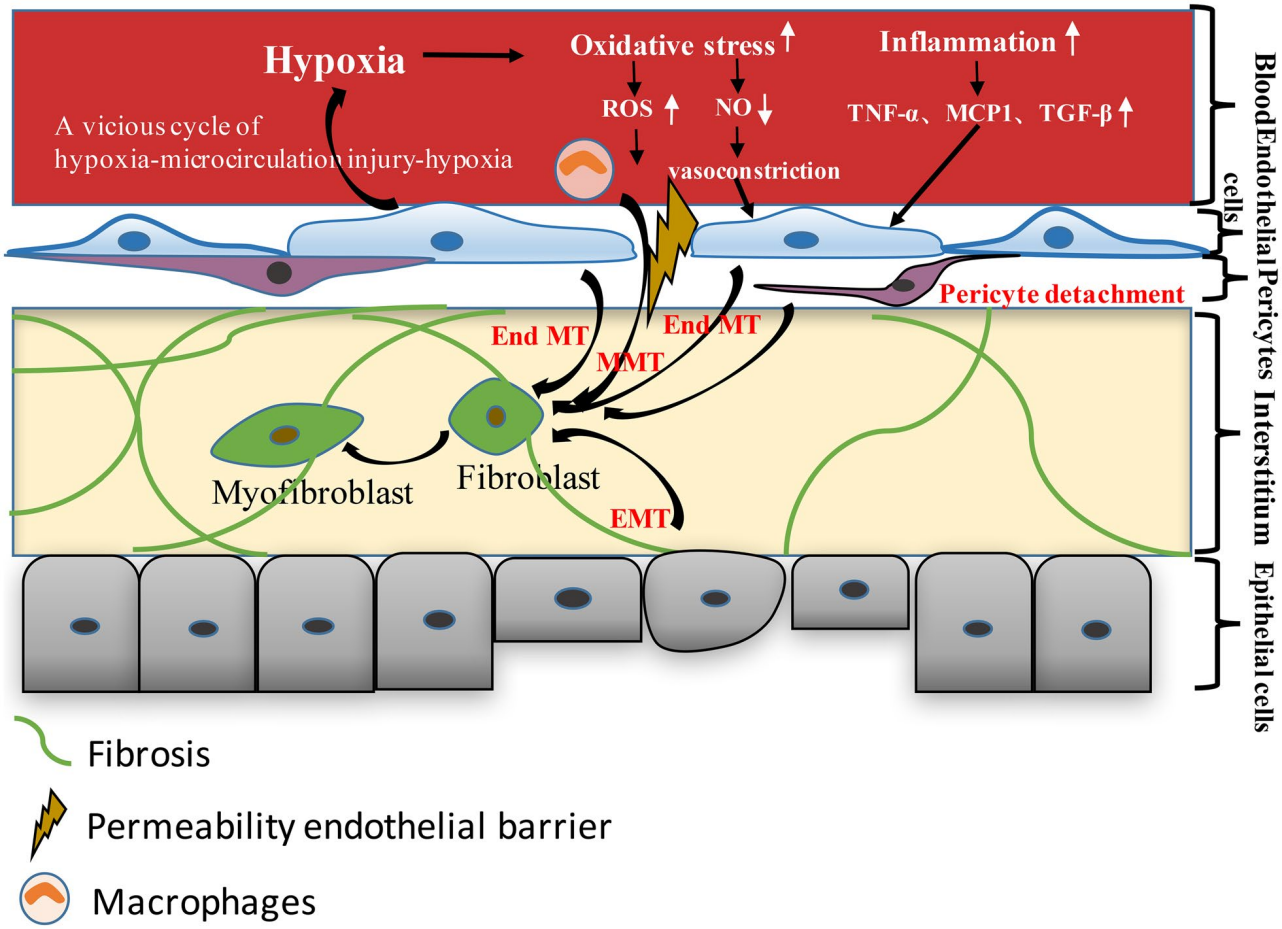
Mechanism underlying the correlation between capillary rarefaction and CKD involves the tissue hypoxia, recruitment of proinflammatory factors and reactive oxygen species (ROS), macrophages infiltration and pericyte detachment and migration. Loss of capillaries accompanied by hypoxia would induce an injury cascade with oxidative stress and inflammation. Proinflammatory cytokines induce not only increase expression of endothelial adhesion molecules but also cells present in the microenvironment, such as fibroblasts and myofibroblasts. Oxidative stress contributes to progression of kidney disease by upregulating production of ROS, which promote apoptosis and necrosis of endothelial cells. Oxidative stress and inflammation also promote endothelial-mesenchymal transition (EndMT) and epithelial-mesenchymal transition (EMT). Macrophages leak into the renal interstitium through injured endothelial cell–cell contacts and undergo macrophage-fibroblast transformation (MMT). The injured endothelial cells induced by stimulus deliver signals to pericytes, leading to the detachment of pericytes from the PTCs, migration into the interstitium, where they differentiate into myofibroblasts

The mechanism underlying the correlation between capillary rarefaction and CKD involves tissue hypoxia, recruitment of proinflammatory factors and reactive oxygen species (ROS), leukocyte adhesion and macrophage infiltration (Fig. 1).

### Hypoxia

In 2002, Fine LG [37] proposed the breathing kidney, emphasizing the role of tubulointerstitial hypoxia accompanied with microvascular damage in progressive kidney disease. We are currently conducting a study that assesses renal hypoxia in patients with CKD using blood oxygenation level-dependent magnetic resonance imaging (BOLD-MRI), the only noninvasive method for evaluating tissue oxygen metabolism. We found that the blood perfusion of renal cortex in CKD patients was less than that in normal controls, indicating that hypoxia is closely related to renal injury. There are many mechanisms of hypoxia-induced renal interstitial fibrosis, most of which have been confirmed: (1) Hypoxia can stimulate the proliferation of fibroblasts, induce their transformation into myofibroblasts and increase extracellular matrix deposition [38, 39]. (2) Hypoxia can stimulate renal parenchyma cells to release protein factors related to renal fibrosis. These protein factors play a significant role in regulating tubulointerstitial fibrosis through paracrine and autocrine pathways [40]. (3) Hypoxia leads to vascular endothelial cell injury and microcirculation disorders, further decrease the availability of oxygen and nutrients for the tissue, and enhance tubular stress and endothelial cell injury, eventually forming a vicious cycle of hypoxia-induced microcirculation injury [41].

### Inflammatory factors

Proinflammatory cytokines increase the expression of endothelial adhesion molecules, including ICAM-1, VCAM-1, and P-selectin in the PTCs [42]. This result enhances endothelium-leukocyte interactions, creating a vigorous proinflammatory state. Moreover, inflammatory activation results in apoptosis of endothelial cells and increase the exposure of endothelial adhesion molecules, which may also lead to impaired microcirculation, causing a positive feedback cycle in the inflammatory response. Some evidence showed that inhibition of the IL-1 receptor signaling molecule IRAK4 results in reduced kidney stromal cell proliferation and fibrosis, highlighting the role of proinflammatory cytokines in the profibrogenic molecular pathway [43].

### Oxidative stress

Oxidative stress is a pathological state induced by an imbalance in the generation and clearance of ROS, which causes lipid peroxidation, increased hydrogen peroxide, and DNA and protein damage. Oxidative stress is elevated because of ischemia-induced hypoxia, which could increase the activity of NADPH oxidases and decrease the release of antioxidant proteins [44, 45]. ROS promote the apoptosis and necrosis of endothelial cells and endothelial cell-to-myofibroblast transition [46, 47] 48. Meanwhile, cell death and inflammation are signals for the recruitment and activation of myofibroblasts, which subsequently cause renal fibrosis [49].

### The immune response

Both innate and adaptive immune responses contribute significantly to the progression of renal injury. Macrophages play an important role in host resistance to infection (innate immunity) and in removing apoptotic cells, cell fragments and foreign bodies. The accumulation of macrophages in the renal interstitium, caused by a damaged endothelial barrier and local proliferation of blood monocytes, is a common feature in most CKD cases. One of the mechanisms of macrophage-mediated fibrosis was elucidated by Neil C. Henderson et al., whom demonstrated that galectin-3 (a β-galactosidase-binding lectin) secreted by macrophages promotes renal fibrosis via a TGF-mediated pathway [50, 51]. Recently, in a study [52] of renal biopsy samples from patients with progressive CKD, coexpression of macrophage markers (F4/80 and CD68) and a fibrotic marker (α-SMA) in conjunction with the accumulation of collagen I were found in the renal interstitium, suggesting that macrophage-to-myofibroblast transition (MMT) may be another pathway that contributes to renal fibrosis in CKD.

### Pericytes

A study conducted by Fligny C and colleagues [53] has identified pericyte detachment from capillaries as a critical step of capillary rarefaction [54]. Pericytes are mesenchymal cells located on the abluminal side of endothelial cells and contribute to the formation and stabilization of microvessels. The injured endothelial cells leads to the delivery of signals to pericytes, following which pericytes detach from the PTCs and migrate into the interstitial space, where they differentiate into myofibroblasts and subsequently contribute directly to pathological matrix deposition [55]. The consequence of this action for microvessels is instability, increased leakage and a damaged basement membrane. Molecular and cellular mechanisms by which pericytes regulate microvascular stability were recently reported [56], identifying TIMP3 as a pericyte factor that improves vascular stability by detecting the loss of TIMP3 during pericyte transition to myofibroblasts. Moreover, TIMP3 has been demonstrated to prevent the activity of some matrix metalloproteinases (MMPs) and a disintegrin and metalloproteinase with thrombospondin motifs-1 (ADAMTS1) and suppress the activation of the endothelial receptor VEGFR2. TIMP3-deficient mice showed more-severe endothelial abnormalities and interstitial fibrosis due to an increase in migration, proliferation and expression of a-SMA and ADAMTS1, which could accelerate capillary tube regression. In a recent study, Kramann R et al. [57] verified their hypothesis that pericyte loss led to tubular injury and PTC rarefaction. Gli1 + pericytes represent a small subfraction of interstitial kidney cells, but a primary source of renal myofibroblasts. After kidney injury, Gli1 + cells detach from microvessel, and ablation of Gli1 + cells induces tissue hypoxia, an inflammatory response, and subsequent sustained capillary rarefaction. Therefore, pericytes might also be a key factor that affects the development of renal fibrosis.

## Methods for assessing microvessels in CKD

Progressive kidney diseases are related to endothelial injury and capillary rarefaction. Therefore, to better understand these pathophysiological processes, tools enabling the quantitative and noninvasive monitoring of vascular changes are required. Microvascular density is usually measured by immunostaining (endothelial cell antigens, such as CD31), estimating the surface area of endothelial cells or counting the number of genetically labeled endothelium (such as Tie 2). However, these methods may lead to errors. Several new technologies have been used to observe tissue microvasculature in recent years (Table 1).

### Synchrotron radiation (SR) for visualizing structures within nephrons

Synchrotron radiation (SR) could help to achieve greater spatial resolution compared with conventional X-ray. Several studies have reported the successful application of SR in angiography. Sonobe T et al. indicated that SR is an effective tool for microangiography of the pulmonary microcirculation (diameters < 100 micron) in the rat [58, 59]. SR contrast angiography has been applied on coronary arteries as small as 100 micron in diameter in a beating heart and 50 micron in an arrested heart [60]. A change in renal microcirculation is closely associated with renal failure. Therefore, SR could be used for investigating structures within nephrons. The SR derived X- rays transmitted through the subjects were recorded by a CCD camera. And the X-rays are converted to visible light on a fluorescent screen. A study of Miya K group showed that the SR renal microangiography (SRRM) was applied to visualize proximal tubules, glomeruli, and renal arterioles, and the minimum diameter of the observed arteriole was 18 μm [61]. A limitation of this method is that all images of nephrons are projected, which do not fully reflect the three-dimensional structures within kidneys.

### Microcomputed tomography (μCT) for anatomical and functional imaging of vascular alterations

μCT for anatomical and functional imaging of microvascular changes could clearly show the branching, size and tortuosity of blood vessels. μCT has already been employed in cancer research, which is characterized by different degrees of angiogenesis. The combination of functional in vivo and anatomical ex vivo X-ray μCT enables a highly precise quantification of relative blood volume and highly detailed analysis of the three-dimensional micromorphology of tumor vessels [62]. Ehling J et al. indicated that the μCT method could accurately reflect hepatic blood vessel alterations in liver fibrosis and become a promising tool for estimating angiogenesis and the effects of antiangiogenic therapy [63]. In renal artery stenosis (RAS) pigs, we have found decreased density of small-size cortical microvessels (< 200 mm) and atherosclerosis-aggravated loss of renal cortical microvessels by using μCT, both of which lead to accelerated renal dysfunction [64]. A research team from Germany used μCT to observe macro-to-microvascular changes in three murine models with different mechanisms of progressive renal disease: the unilateral ureteral obstruction (UUO) model, ischemia–reperfusion (I/R) injury model, and progressive glomerulonephritis model (Alport mice), demonstrating the close correlation between PTC rarefaction and kidney injury. Mice were imaged before and immediately after intravenous injection of 100 ml eXIATM160XL, which is an iodine-based contrast agent optimized for in vivo μCT-based blood pool imaging,This method provide a noninvasive visualization of renal vessels with a spatial resolution of 35 mm voxel side length [30]. However, there are some limitations to this method, including X-ray exposure and the need for iodine-based contrast agents [65].

### FMA method for assessing the microvascular architecture of the kidney

**Fig. 2:**
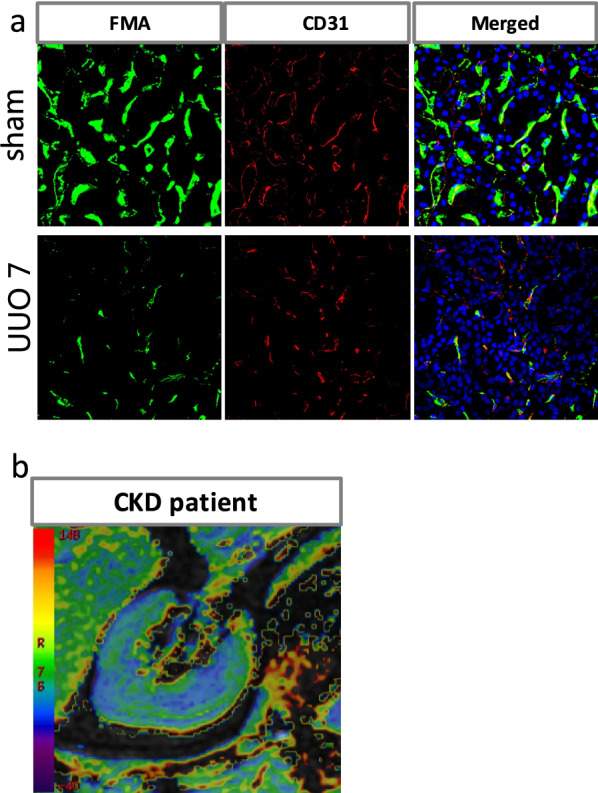
**a** The fluorescent microangiography (FMA) method is based on low-melting-point agarose with fluorescent polystyrene microspheres which could perfuse the microvasculature and identified a decrease in both glomerular and PTC density in the kidneys of UUO model. Immunostaining surrounding with CD31 (expressed on the surface of endothelial cells) demonstrates capillary rarefaction in response to the obstructed kidney. **b** Renal R2* images in the CKD patient: Uneven blue in the cortex indicates decreased cortical oxygenation. The large green, yellow and red areas in the medulla indicate decreased medullary oxygenation

Recently, Advani A and colleagues [66] reported the use of fluorescence microangiography (FMA) by renal artery injection in 5/6 nephrectomy mice. The FMA method allowed the three-dimensional reconstruction of renal microvasculature and circumvents and identified a decrease in both glomerular and PTC density in subtotally nephrectomized rats. FMA uses low-melting-point agarose with fluorescent polystyrene microspheres, which could perfuse the microvasculature and provide a microangiogram via confocal microscopy. The FMA method provides an optimal structure of the renal microvessels and avoids limitations of endothelial antigens, which may be coexpressed by the lymphatic vasculature. Moreover, a team from Harvard Medical School improved this technique by intracardiac injection, allowing a highly distinct visualization of vascular perfusion in the kidney, heart and liver. Additionally, the team created a MATLAB-based script that allows the precise analysis of totally perfused cortices, capillary number and even the perimeter and area of the individual capillary cross-section. These researchers found that the loss of peritubular perfusion and capillary density in the AKI model was closely correlated with the severity of renal injury and further leads to renal interstitial fibrosis [29]. In our recently study, we applied FMA method to evaluate the renal microvasculature and found that the capillary number and total perfused area were reduced in the UUO 7d group compared with the sham group. Additionally, in order to mark the outline of PTCs, CD31, an endothelial cell antigens expressed on endothelial cell surface was utilized, and showed that capillaries surrounded by CD31 + endothelial cells in the UUO group appeared a decreased luminal FMA signal, indicating a lack of perfusion of these capillary vessels [67] (Fig. 2a).

### Blood Oxygen Level- dependent Magnetic Resonance Imaging (BOLD-MRI) for accessing tissue oxygen metabolism

BOLD-MRI, which is based on paramagnetic properties of deoxyhemoglobin, could evaluate the degree of tissue hypoxia by measuring the deoxyhemoglobin level. BOLD-MRI is also the only noninvasive, safe and reliable method to access tissue oxygen metabolism [68]. BOLD-MRI is primarily used in brain functional imaging. With advances in scanning technology, BOLD-MRI has been applied more widely in kidney diseases, including I/R renal injury [69], diabetic nephrology [70], contrast nephropathies [71] and renal allograft rejection [72]. Renal diseases are often accompanied by changes in microvascular and renal perfusion, which further induce hypoxia in tissue. Therefore, changes in the level of oxygenation in the kidney can often reflect abnormal changes in the early stage of the disease and indirectly evaluate the microcirculation of the tissues. A research team from the Mayo Clinic explored the relationship between renal blood oxygen level and renal blood perfusion in patients with renal vascular diseases before and after the use of diuretics by using BOLD-MRI. This team found that the decrease in blood flow and GFR were closely related to the fractional hypoxia of kidney tissue and that a diuretic could improve renal hypoxia [73]. In a study from our group, we used regional and whole-kidney ROI selection methods combined with the 3.0-T MRI creatively to evaluate the renal oxygenation in CKD patients. This system automatically generated an R2* pseudocolor map showing a gradual transition from blue (represents high renal tissue oxygenation) to green, yellow and red (represents significant hypoxia), indicating a gradual increase in renal hypoxia. The results showed decreased cortical and medullary oxygenation compared with the healthy people [74] (Fig. 2b). In addition, BOLD-MRI avoids the use of exogenous contrast agent, which is a great advantage for clinical application.

### Electron microscopy for visualizing subtle ultrastructure of the endothelium

Normal peritubular capillary endothelial cells are thin or flat with regular contours. The cell soma of endothelial cells is perforated by fenestrations, which make the cytoplasm travel in small segments. Electron microscopy is the only method to visualize subtle ultrastructure, indicating that the very early changes of endothelial cells could be detected. Babickova J et al. [9] have studied the ultrastructural alterations of the microvasculature in 3 murine models of different kidney disease. Compared with the kidneys of control group, decreased numbers of fenestrations, the widening of the subendothelial space and increased numbers of endothelial vacuoles and caveolae were detected by Electron microscopy in fibrotic kidneys. These findings allow us to visualize the damage of endothelial cells of PTCs in progressive kidney disease and is in line with recent data showing the role of microvascular endothelium in renal intestitial fibrosis [75]. However, the limitation to this method is the potential sampling error, because only a very small amount of tissue is analyzed.

### Intravital multiphoton microscopy (MPM) for the single-cell–level investigation

MPM is a powerful technique to study alterations in tissue morphology and function simultaneously in the living animal, which perform long-term cell fate tracing of the same renal cells by following ex vivo histology [76]. Completely noninvasive and high-quality renal imaging can be achieved through an abdominal imaging window (AIW), which consists of coverslipped titanium ring, adhering to the kidney. This enables researchers to obtain structural and functional data of individual nephron in the same region within a certain period of time, avoids the problem of heterogeneity of renal tissue, and establishes the dynamics and patterns of individual cell’ movement and migration over time. Schiessl IM's group [77] have successfully use this method to track the migration and differentiation of individual renal progenitor cell in homozygous Ren1d-Confetti reporter mice. Schiessl IM et al. [78] have investigated tubular cells migrate and proliferate to the location of ablated tubular cells and reconstitute the functional integrity of the tubule by using MPM with subsequent ex vivo histology. Therefore, we believe that this new technique is hopeful to be applied to the dynamic observation of renal endothelial cells and renal interstitial cells, and cell-to-cell communication with functional alterations in vivo.

## Therapeutic strategies targeting injured microvessels

CKD is characterized by renal fibrosis, with excessive accumulation of extracellular matrix leading to progressive renal failure. Mounting evidence has demonstrated the significant role of microvasculature injury in the development of interstitial fibrosis and CKD progression. Therefore, restoration of renal microcirculation is a rapidly expanding area of research and a promising therapy for the prevention of kidney fibrosis. Unfortunately, despite current treatments, the majority of patients still experience progressive renal failure and develop ESRD. To solve these conventional therapeutic limitations, new and affordable therapies are urgently needed.

### Growth factors and cytokines

Several growth factors and cytokines have been reported to ameliorate renal fibrosis in experimental models of CKD (Table 2).

**Table 1:** Methods of accessing microvessels in CKD

Methods	Applications	Advantages	Disadvantages	References
Synchrotron radiation (SR)	Visualizing proximal tubules, glomeruli and renal arterioles	Non-invasive method; visualizing the smaller arteriole	Can not fully reflect the three-dimensional structures within kidneys	[61]
Micro-computed tomography (μCT)	Quantitative monitoring for anatomical and functional changes of vascular alterations	Non-invasive method; user- friendliness; operator independence; quantitative assessment	X-ray exposure; the need for iodine- based contrast agents;	[30, 65]
FMA-method	Assessing the microvascular architecture of the kidney	Enabled the three-dimensional reconstruction; quantitative assessment	Sacrifice of the animals	[29, 67]
BOLD-MRI	Accessing tissue oxygen metabolism and indirectly evaluating the microcirculation	Noninvasive method; without exposure to radiation or exogenous contrast agents; high spatial resolution	The R2* value is easily affected; the absence of cortico- medullary differentiation	[74]
Electron microscopy	Visualizing subtle ultrastructure of the endothelium	Visualizing the very early changes to endothelium	Sacrifice of the animals; the potential sampling error	[9]
Intravital Multiphoton Microscopy (MPM)	Quantitative evaluations of numerous renal functions	Noninvasive; high-resolution	Motion artifact; endogenous autofluorescence; long time image collection	[77, 78]

**Table 2:** Growth factors and cytokines for improving renal microcirculation

Setting	Intervention	Mechanism	Effects	References
Chronic renovascular desease (unilateral renal artery stenosis)	Vascular endothelial growth factor(VEGF)	Restored microvascular density	Attenuated fibrogenic activity and improved kidney blood flow and GFR	[81]
Renal interstitial fibrosis (monocrotline treated rats)	Glucagon-Like Peptide-1(GLP)	Reduced microcirculation lesion and inhibit EndMT	Reduced microcirculation lesion-induced renal fibrosis	[82]
Renal interstitial fibrosis (UUO model)	Angiopoietin-1 (Ang-1)	Stimulated the growth of peritubular capillaries and dampened renal inflammation	Attenuated myofibroblast activation and interstitial collagen I accumulation	[83]
Renal interstitial fibrosis (UUO model)	Insulin-Like Growth Factor- 1 (IGF-1R)	Preserved endothelial function by stabilization of the VE-protein tyrosine phosphatase/VE-cadherin complex	Suppressed the inflammatory cell infiltration and renal fibrosis	[85]
Chronic kidney disease (5/6 nephrectomized rats)	Netrin-1	inhibited EndMT	Attenuated the progression of renal dysfunction	[87]

VEGF has shown promise in improving renal function in various models of renal disease. Tissue hypoxia induced by chronic ischemia not only leads to significant microvascular injury but also an impaired angiogenic response that results in decreased expression of VEGF and altered downstream signaling pathways[67, 79, 80]. Based on these findings, VEGF is regarded as one of the promising therapeutic growth factors for microcirculation improvement and is well known as the most potent inducer of angiogenesis and an endogenous regulator of endothelial integrity. Chade AR et al. demonstrated that intrarenal VEGF therapy significantly reduced microcirculation injury and restored renal function in a swine model of RAS through the induction of proliferation, endothelial tube formation, and migration of endothelial cells. However, as VEGF is limited by its short t1/2 and its susceptibility to degradation, a biopolymer-stabilized elastin-like polypeptide (ELP)-VEGF fusion protein was generated to improve the bioavailability of VEGF. The study indicated that renoprotective effects after ELP-VEGF therapy was largely driven by modulation of renal macrophages toward the anti-inflammatory M2 phenotype, restoring VEGF signaling and sustaining improvement of renal function and microvascular integrity in CKD [81].

A group of Xu J demonstrated that GLP-1R agonist have a protective effect against renal interstitial fibrosis induced by renal microcirculation injury. Activating GLP-1R resulted in positive effect via antiapoptotic effect, inhibiting the phenotypic conversion of renal microvascular vascular smooth muscle cells, and inhibiting endothelial-to-mesenchymal transition (EndMT) through increasing BMPR2 and decreasing TGF- β1 [82].

Angiopoietin-1 (Ang-1), a vascular growth factor secreted by renal proximal tubular cells and podocytes, has endothelial network formation and potent EC sprouting and migration capacity. Ang-1-deficient mice have been reported to exhibit severe organ damage, and fibrosis was promoted via the regulation of vascular density. Moreover, Singh S showed a potential therapeutic effect of Ang-1 by activating Tie2 to inhibit TNFα-induced leukocyte capillary transmigration and ultimately repairing microvasculature damage [83].

Insulin-like growth factor-1 receptor (IGF-1R), a member of the tyrosine kinase receptor superfamily, is a transmembrane receptor. IGF-I and IGF-II receptors are abundantly expressed on endothelial cells, demonstrating that these growth factors play a physiological role in microvessels [84]. IGF-1R contributes to the regulation of cellular proliferation, differentiation, and survival. To demonstrate the critical role of IGF-1R in maintaining endothelial function, Liang M et al. established IGF-1R KO mice and detected increased interstitial fibrosis and enhanced extracellular matrix (ECM) deposition. Conversely, inflammation and renal fibroblast induced by UUO were mitigated after IGF-1R overexpression by reducing vascular hyperpermeability [85].

Netrin-1 is a laminin-related secreted protein that could play a critical role in promoting vascular endothelial cell migration and angiogenesis. Several studies have reported the protective effect of netrin-1 on kidney diseases, including I/R renal injury and diabetic nephropathy [86]. Therefore, Bai J et al. explored the potential therapeutic effect of netrin-1 on CKD. Animal experiments were performed to demonstrate that netrin-1 could alleviate the progression of renal failure and attenuate interstitial fibrosis by suppressing EndMT in 5/6 nephrectomy rats via blockade of the TGF-β/Snail/α-SMA signaling pathway [87].

### Cell therapy

Cell-based therapy is not only the most advanced regenerative therapeutic strategy to date but one that has shown great promise for the treatment of CKD [88]. The most widely used cell is the stem cell, given its ability to be used in animal models of kidney diseases [89, 90]. By targeting the underlying mechanism of the renal diseases, stem cell-based therapy restores the structure and functionality of damaged tissues by stimulating the endogenous regenerative capacity [91]. There are different types of stem cells available for regenerative therapy (Table 3). Embryonic stem cells (ESCs) and human induced pluripotent stem cells (hiPSCs) are those with the most differentiation potency. However, these cells also seem to have the greatest tumorigenesis property. Van den Berg et al. [92] demonstrated that hiPSC-derived kidney organoids develop host-derived vascularization in vivo and contribute to the formation of a glomerular basement membrane and fenestrated endothelial cells as detected by ultrastructural evaluation. Musah et al. [93] showed that hiPSCs had a remarkable capacity to self-renew indefinitely and differentiate into endothelial cells by coculture with human kidney glomerular endothelium. Despite their potent differentiation capacity, the widespread use of ESCs and hiPSCs are limited by ethical issues and high tumorigenicity [94].

**Table 3:** Cell-based therapy for improving renal microcirculation

Setting	Intervention	Mechanism	Effects	references
Renal interstitial fibrosis (UUO model)	Bone marrowderived endothelial progenitor cells (BM-EPCs)	Increased the number of capillary density	Alleviated interstitial fibrosis	[97]
Renal interstitial fibrosis (UUO model)	Adipose-derived mesenchymal stemcells (AMSCs)	Improved renal microcirculation through PI3K/Akt/eNOS signaling pathway and inhibited the EndMT process	Inhibited the kidney fibrogenesis	[101]
AKI (ischeamia/reperfusion I/R model)	Human adipose stromal cells (hASCs)	Decreased inflammation and preserved peritubular capillaries	Improved recovery from I/R- induced injury and chronic kidney injury	[102]
AKI (ischeamia/reperfusion I/R model)	Bone marrow mesenchymal stromal cells (BMSCs)	Protected microvasculature and strongly suppressed fibrosis via the effects of vasoprotective cytokines	Suppressed progressive fibrosis in kidney diseases	[104]
Chronic renal failure (adenine induced CRF model)	BMSCs	Increased the glomerular capillaries density and promoted the secretion of VEGF via activating PI3k-Akt signaling pathway	Improved renal function	[105]
Renal interstitial fibrosis (unilateral ureteral obstruction UUO model)	Human amniotic fluid-derived stem cells (hAFSCs)	increased in microvascular density and relieve hypoxia	Alleviated interstitial fibrosis alleviated interstitial fibrosis	[106]

Endothelial progenitor cells (EPCs), which are derived from the bone marrow, play a critical role in repairing injured microvessels and promoting angiogenesis. Chen et al. reported that CKD patients showed lower EPC numbers in circulation compared with heathy controls, which is associated with cell apoptosis resulting from increased oxidative stress and correlated to defective neovascularization and endothelial dysfunction [95]. Based on their capacity to regenerate endothelium, EPCs are considered a promising therapeutic agent for CKD. Intrarenal infusion of autologous EPCs has been reported to improve renal microcirculation in chronic experimental renovascular disease. Microvascular density and vascular volume were increased after the administration of EPCs, as detected directly by μCT. EPCs could not only generate new vessels but also promote the stabilization and maturation of the new vessels, resulting in renal protection [96]. In our previous study, bone marrow-derived EPC transplantation stimulated the release of angiogenic cytokines and increased PTC density. Therefore, blood supply to renal tubules and oxygen delivery were improved, leading to further alleviation of renal interstitial fibrosis in obstructive nephropathy in mice [97]. The mechanism by which EPCs preserve capillaries and promote angiogenesis involves homing to the injured kidney and paracrine secretion of growth factors [4, 98].

Mesenchymal stem cells (MSCs) are the most widely used cells in regenerative medicine therapy of kidney diseases [99]. To date, approximately 1068 active clinical trials investigating the potential of MSCs are listed in the US National Library of Medicine (http://clinicaltrials.gov, Mar 2020). These clinical trials span a broad range of applications, such as in the treatment of tumors, diabetes mellitus, sexual dysfunction, hematological diseases, ischemic injuries, and immune disorders (including autoimmune urticarial, lupus, systemic scleroderma, Crohn’s disease, and graft-versus-host disease) and in soft tissue regeneration. Among these trials, 33 are investigating MSC targeting in kidney diseases. A research team from the Mayo Clinic has completed a clinical trial on 14 atherosclerotic renovascular disease (RVD) patients and demonstrated the safety and efficacy of intraarterial injection of autologous adipose-derived MSCs [100]. MSC infusion could alleviate renal tissue hypoxia and increase the cortical blood supply to ameliorate the progression of renal failure. Several studies have recently shown that MSCs play an important role in preserving renal microvessels and relieving kidney injury in a variety of kidney disease models (Table 3). Furthermore, MSCs can be obtained from a variety of tissues, including adipose tissue [101, 102], bone marrow [103–105] and amniotic fluid [106, 107], and have been reported to have antioxidative, anti-inflammation, proangiogenic, and immunomodulatory function in numerous experimental studies. More specifically, the functions (including differentiation, immunosuppression, and release of cytokines and factors) of MSCs isolated from adipose tissue of renal disease patients were not affected by exposure to uremic conditions. These results indicate the feasibility of developing therapies with autologous MSCs for renal failure patients [108].

### Kidney‐specific intervention by targeting genes

Several studies have demonstrated that some genes play a significant role in renal injury by detecting their high expression in different animal models (Table 4). Therefore, inhibition of specific genes is considered a potential therapeutic approach. Deng et al. [109] found that protein phosphatase 2A (PP2A) is significantly activated by TGF-β1 stimulation, but endothelial integrity and function are restored after inhibiting PP2A activation, thereby preventing the progression of endothelial cell differentiation into scar-forming myofibroblasts. A study from Stuart W S showed that the genetic loss of CD248, a type I transmembrane glyco-protein that is expressed by stromal fibroblasts and myofibroblasts, could protect against myofibroblast accumulation through alleviating microvascular rarefaction and modulation of pericyte and stromal cell function [110]. There is increasing evidence that EGFR activation plays an important role in the initiation and progression of renal disease. EGF deletion has been reported to have a protective role in acute kidney injury [111], glomerular injury [112] and diabetic nephropathy [113]. Furthermore, Zeng et al. demonstrated that endothelial deletion of heparin-binding EGF-like factor (HB-EGF) attenuated endothelial cell apoptosis, resulting in the protection of glomerular capillary integrity and, consequently, the alleviation of renal fibrosis [114]. Our previous study also found that a specific gene, thrombospondin-1 (TSP-1), was highly expressed in the interstitium of both experimental [115] and human [116] interstitial fibrosis and that suppression of TSP-1 increased VEGF levels and PTC density. These results suggested that TSP-1 gene silencing prevented renal fibrosis and may become a new potential therapy for CKD. Recent evidence indicates that long non-coding RNA (lncRNA) is rapidly emerging as a promising drug targeting angiogenesis. Knock- down of lncRNA MALAT1 is reported to inhibit high glucose induced-fibrosis in the diabetic nephropathy model, which may due to the mechanism of supressing endothelial to mesenchymal transition via targeting miR-145/ZEB2 axis. These results provide a novel potential target for renal disease in the future [117]. Although many promising therapeutic strategies aiming to silence endothelial genes and restore renal microcirculation have been confirmed to be effective in animal models, to date, there is still a lack of evidence from clinical trials.

**Table 4:** Kidney-specific intervention targeting genes for improving renal microcirculation

Setting	Intervention	Mechanism	Effects	References
Renal interstitial fibrosis (UUO model)	Protein phosphatase 2A knockout	Preserved density of peritubular capillaries and ameliorated renal EndoMT level	Prevented renal fibrosis	[109]
Renal interstitial fibrosis (UUO model)	Deletion of the Stromal Cell Marker CD248 (Endosialin)	Inhibited microvascular rarefaction through modulation of pericyte and stromal cell function	Ameliorated renal fibrosis	[110]
Chronic kidney disease (Ang-II induced renal injury)	Deletion of heparinbinding EGF-like factor (HB-EGF)	Preserved endothelial integrity and reduced inflammation in the perivascular area	Ameliorated renal injury	[114]
Renal interstitial fibrosis (UUO model)	Thrombospondin-1 (TSP-1) gene silencing	Increased PTC density and ameliorate hypoxia	Prevented renal fibrosis	[115]
Diabetic nephropathy (db/db model of DN)	lncRNA-MALAT1 knockout	Repressed endothelialto-mesenchymal transition via targeting miR-145/ZEB2 axis	Inhibited HGinduced fibrosis	[117]

## Conclusion

The endothelium is considered a key contributor to the maintenance of normal vascular homeostasis, and injured endothelial cells lead to progressive fibrosis and loss of renal function. A number of cellular mechanisms are involved, including tissue hypoxia, inflammatory cell infiltration, oxidative stress, the immune response, pericyte detachment and EndoMT. Techniques for assessing renal microcirculation will help acquire a better understanding of the role of capillary rarefaction in CKD and direct therapeutic strategies to restoring capillaries. Based on the close relationship between microvascular damage and kidney diseases, novel therapeutic options targeted toward endothelium have become promising therapies and are thus expected to become effective clinical treatments for patients with CKD. Science and technology development fund of Affiliated Hospital of Xuzhou Medical University (XYFC2020001; XYFY2020038).

## Data Availability

Not applicable.

## Abbreviations

CKD: Chronic kidney disease
ESRD: End-stage renal disease
NO: Nitric oxide
eNOS: Endothelial NO synthase
RAS: Renin–angiotensin system
Ang-2: Angiopoietin-2
VEGF: Vascular endothelial growth factor
PTC: Peritubular capillary
AKI: Acute kidney injury
FMA: Fluorescence microangiography
SHRs: Spontaneous hypertensive rats
HUVECs: Umbilical vein endothelial cells
BOLD-MRI: Blood oxygenation level-dependent magnetic resonance imaging
MMT: Macrophage-to-myofibroblast transition
MMPs: Matrix metalloproteinases
SR: Synchrotron radiation
MPM: Multiphoton microscopy
AIW: Abdominal imaging window
ECM: Enhanced extracellular matrix
EndoMT: Endothelial-mesenchymal transition
ESCs: Embryonic stem cells
hiPSCs: Human induced pluripotent stem cells
EPCs: Endothelial progenitor cells
MSCs: Mesenchymal stem cells
RVD: Renovascular disease
HB-EGF: Heparin-binding EGF-like factor
TSP-1: Thrombospondin-1

## References

[1] Fraser SD, Blakeman T (2016). Chronic kidney disease: identification and management in primary care. Pragmat Obs Res.

[2] Zhang L, Wang F, Wang L, Wang W, Liu B, Liu J, Chen M, He Q, Liao Y, Yu X (2012). Prevalence of chronic kidney disease in China: a cross-sectional survey. Lancet.

[3] Flisinski M, Brymora A, Bartlomiejczyk I, Wisniewska E, Golda R, Stefanska A, Paczek L, Manitius J (2012). Decreased hypoxia-inducible factor-1alpha in gastrocnemius muscle in rats with chronic kidney disease. Kidney Blood Press Res.

[4] Prommer HU, Maurer J, von Websky K, Freise C, Sommer K, Nasser H, Samapati R, Reglin B, Guimaraes P, Pries AR (2018). Chronic kidney disease induces a systemic microangiopathy, tissue hypoxia and dysfunctional angiogenesis. Sci Rep.

[5] Amann K, Wiest G, Zimmer G, Gretz N, Ritz E, Mall G (1992). Reduced capillary density in the myocardium of uremic rats–a stereological study. Kidney Int.

[6] Tornig J, Amann K, Ritz E, Nichols C, Zeier M, Mall G (1996). Arteriolar wall thickening, capillary rarefaction and interstitial fibrosis in the heart of rats with renal failure:the effects of ramipril, nifedipine and moxonidine. J Am Soc Nephrol.

[7] Burns CM, Knopman DS, Tupper DE, Davey CS, Slinin YM, Lakshminarayan K, Rossom RC, Pederson SL, Gilbertson DT, Murray AM (2018). Prevalence and risk of severe cognitive impairment in advanced chronic kidney disease. J Gerontol A Biol Sci Med Sci.

[8] Ohashi R, Shimizu A, Masuda Y, Kitamura H, Ishizaki M, Sugisaki Y, Yamanaka N (2002). Peritubular capillary regression during the progression of experimental obstructive nephropathy. J Am Soc Nephrol.

[9] Babickova J, Klinkhammer BM, Buhl EM, Djudjaj S, Hoss M, Heymann F, Tacke F, Floege J, Becker JU, Boor P (2017). Regardless of etiology, progressive renal disease causes ultrastructural and functional alterations of peritubular capillaries. Kidney Int.

[10] Cui J, Wu X, Song Y, Chen Y, Wan J (2019). Complement C3 exacerbates renal interstitial fibrosis by facilitating the M1 macrophage phenotype in a mouse model of unilateral ureteral obstruction. Am J Physiol Renal Physiol.

[11] Guise E, Engel JE, Williams ML, Mahdi F, Bidwell GL, Chade AR (2019). Biopolymer-delivered vascular endothelial growth factor improves renal outcomes following revascularization. Am J Physiol Renal Physiol.

[12] Sun IO, Santelli A, Abumoawad A, Eirin A, Ferguson CM, Woollard JR, Lerman A, Textor SC, Puranik AS, Lerman LO (2018). Loss of renal peritubular capillaries in hypertensive patients is detectable by urinary endothelial microparticle levels. Hypertension.

[13] Vanhoutte PM, Shimokawa H, Feletou M, Tang EH (2017). Endothelial dysfunction and vascular disease—a 30th anniversary update. Acta Physiol (Oxf).

[14] Aird WC (2012). Endothelial cell heterogeneity. Cold Spring Harb Perspect Med.

[15] Eelen G, de Zeeuw P, Simons M, Carmeliet P (2015). Endothelial cell metabolism in normal and diseased vasculature. Circ Res.

[16] Toblli JE, DiGennaro F, Giani JF, Dominici FP (2012). Nebivolol: impact on cardiac and endothelial function and clinical utility. Vasc Health Risk Manag.

[17] Zhi Z, Pengfei Z, Xiaoyi T, Genshan M (2014). Adiponectin ameliorates angiotensin II-induced vascular endothelial damage. Cell Stress Chaperones.

[18] Yang D, Zhang M, Huang X, Fang F, Chen B, Wang S, Cai J, Shi X, Qu J, Geng YJ (2010). Protection of retinal vasculature by losartan against apoptosis and vasculopathy in rats with spontaneous hypertension. J Hypertens.

[19] Stow LR, Jacobs ME, Wingo CS, Cain BD (2011). Endothelin-1 gene regulation. FASEB J.

[20] Rathnakumar K, Savant S, Giri H, Ghosh A, Fisslthaler B, Fleming I, Ram U, Bera AK, Augustin HG, Dixit M (2016). Angiopoietin-2 mediates thrombin-induced monocyte adhesion and endothelial permeability. J Thromb Haemost.

[21] Raees A, Bakhamis A, Mohamed-Ali V, Bashah M, Al-Jaber M, Abraham D, Clapp LH, Orie NN (2019). Altered cyclooxygenase-1 and enhanced thromboxane receptor activities underlie attenuated endothelial dilatory capacity of omental arteries in obesity. Life Sci.

[22] Stanhewicz AE, Alexander LM (2020). Local angiotensin-(1–7) administration improves microvascular endothelial function in women who have had preeclampsia. Am J Physiol Regul Integr Comp Physiol.

[23] Yan M, Hu Y, Yao M, Bao S, Fang Y (2017). GM-CSF ameliorates microvascular barrier integrity via pericyte-derived Ang-1 in wound healing. Wound Repair Regen.

[24] Hastie R, Tong S, Hannan NJ, Brownfoot F, Cannon P, Kaitu'u-Lino TJ (2017). Epidermal growth factor rescues endothelial dysfunction in primary human tissues in vitro. Reprod Sci.

[25] Davis S, Aldrich TH, Jones PF, Acheson A, Compton DL, Jain V, Ryan TE, Bruno J, Radziejewski C, Maisonpierre PC (1996). Isolation of angiopoietin-1, a ligand for the TIE2 receptor, by secretion-trap expression cloning. Cell.

[26] Linares PM, Chaparro M, Gisbert JP (2014). Angiopoietins in inflammation and their implication in the development of inflammatory bowel disease. A review. J Crohns Colitis.

[27] Maisonpierre PC, Suri C, Jones PF, Bartunkova S, Wiegand SJ, Radziejewski C, Compton D, McClain J, Aldrich TH, Papadopoulos N (1997). Angiopoietin-2, a natural antagonist for Tie2 that disrupts in vivo angiogenesis. Science.

[28] Vestweber D (2012). Relevance of endothelial junctions in leukocyte extravasation and vascular permeability. Ann N Y Acad Sci.

[29] Kramann R, Tanaka M, Humphreys BD (2014). Fluorescence microangiography for quantitative assessment of peritubular capillary changes after AKI in mice. J Am Soc Nephrol.

[30] Ehling J, Babickova J, Gremse F, Klinkhammer BM, Baetke S, Knuechel R, Kiessling F, Floege J, Lammers T, Boor P (2016). Quantitative micro-computed tomography imaging of vascular dysfunction in progressive kidney diseases. J Am Soc Nephrol.

[31] Advani A, Gilbert RE (2012). The endothelium in diabetic nephropathy. Semin Nephrol.

[32] Maric-Bilkan C, Flynn ER, Chade AR (2012). Microvascular disease precedes the decline in renal function in the streptozotocin-induced diabetic rat. Am J Physiol Renal Physiol.

[33] Bidani AK, Polichnowski AJ, Loutzenhiser R, Griffin KA (2013). Renal microvascular dysfunction, hypertension and CKD progression. Curr Opin Nephrol Hypertens.

[34] Brenner BM, Lawler EV, Mackenzie HS (1996). The hyperfiltration theory: a paradigm shift in nephrology. Kidney Int.

[35] Hao HF, Liu LM, Pan CS, Wang CS, Gao YS, Fan JY, Han JY (2017). Rhynchophylline ameliorates endothelial dysfunction via Src-PI3K/Akt-eNOS cascade in the cultured intrarenal arteries of spontaneous hypertensive rats. Front Physiol.

[36] Kang DH (2018). Hyperuricemia and progression of chronic kidney disease: role of phenotype transition of renal tubular and endothelial cells. Contrib Nephrol.

[37] Fine LG, Norman JT (2002). The breathing kidney. J Am Soc Nephrol.

[38] Liu M, Ning X, Li R, Yang Z, Yang X, Sun S, Qian Q (2017). Signalling pathways involved in hypoxia-induced renal fibrosis. J Cell Mol Med.

[39] Liu K, Fang C, Shen Y, Liu Z, Zhang M, Ma B, Pang X (2017). Hypoxia-inducible factor 1a induces phenotype switch of human aortic vascular smooth muscle cell through PI3K/AKT/AEG-1 signaling. Oncotarget.

[40] Liu J, Wei Q, Guo C, Dong G, Liu Y, Tang C, Dong Z (2017). Hypoxia, HIF, and associated signaling networks in chronic kidney disease. Int J Mol Sci.

[41] Zafrani L, Ince C (2015). Microcirculation in acute and chronic kidney diseases. Am J Kidney Dis.

[42] Wu X, Guo R, Wang Y, Cunningham PN (2007). The role of ICAM-1 in endotoxin-induced acute renal failure. Am J Physiol Renal Physiol.

[43] Lemos DR, McMurdo M, Karaca G, Wilflingseder J, Leaf IA, Gupta N, Miyoshi T, Susa K, Johnson BG, Soliman K (2018). Interleukin-1beta activates a MYC-dependent metabolic switch in kidney stromal cells necessary for progressive tubulointerstitial fibrosis. J Am Soc Nephrol.

[44] Chao CT, Chiang CK (2015). Uremic toxins, oxidative stress, and renal fibrosis: an interwined complex. J Ren Nutr.

[45] Schmidt HH, Stocker R, Vollbracht C, Paulsen G, Riley D, Daiber A, Cuadrado A (2015). Antioxidants in translational medicine. Antioxid Redox Signal.

[46] Massberg S, Enders G, Leiderer R, Eisenmenger S, Vestweber D, Krombach F, Messmer K (1998). Platelet-endothelial cell interactions during ischemia/reperfusion: the role of P-selectin. Blood.

[47] Patschan D, Schwarze K, Henze E, Becker JU, Patschan S, Muller GA (2014). eEOC-mediated modulation of endothelial autophagy, senescence, and EnMT in murine diabetic nephropathy. Am J Physiol Renal Physiol.

[48] Li J, Qu X, Bertram JF (2009). Endothelial-myofibroblast transition contributes to the early development of diabetic renal interstitial fibrosis in streptozotocin-induced diabetic mice. Am J Pathol.

[49] Liles JT, Corkey BK, Notte GT, Budas G, Lansdon EB, Hinojosa-Kirschenbaum F, Badal SS, Lee M, Schultz BE, Wise S (2018). ASK1 contributes to fibrosis and dysfunction in models of kidney disease. J Clin Invest.

[50] Henderson NC, Mackinnon AC, Farnworth SL, Kipari T, Haslett C, Iredale JP, Liu FT, Hughes J, Sethi T (2008). Galectin-3 expression and secretion links macrophages to the promotion of renal fibrosis. Am J Pathol.

[51] Meng XM, Nikolic-Paterson DJ, Lan HY (2014). Inflammatory processes in renal fibrosis. Nat Rev Nephrol.

[52] Wang YY, Jiang H, Pan J, Huang XR, Wang YC, Huang HF, To KF, Nikolic-Paterson DJ, Lan HY, Chen JH (2017). Macrophage-to-myofibroblast transition contributes to interstitial fibrosis in chronic renal allograft injury. J Am Soc Nephrol.

[53] Fligny C, Duffield JS (2013). Activation of pericytes: recent insights into kidney fibrosis and microvascular rarefaction. Curr Opin Rheumatol.

[54] Tanaka S, Tanaka T, Nangaku M (2015). Hypoxia and dysregulated angiogenesis in kidney disease. Kidney Dis (Basel).

[55] Lin SL, Kisseleva T, Brenner DA, Duffield JS (2008). Pericytes and perivascular fibroblasts are the primary source of collagen-producing cells in obstructive fibrosis of the kidney. Am J Pathol.

[56] Schrimpf C, Xin C, Campanholle G, Gill SE, Stallcup W, Lin SL, Davis GE, Gharib SA, Humphreys BD, Duffield JS (2012). Pericyte TIMP3 and ADAMTS1 modulate vascular stability after kidney injury. J Am Soc Nephrol.

[57] Kramann R, Wongboonsin J, Chang-Panesso M, Machado FG, Humphreys BD (2017). Gli1(+) pericyte loss induces capillary rarefaction and proximal tubular injury. J Am Soc Nephrol.

[58] Sonobe T, Schwenke DO, Pearson JT, Yoshimoto M, Fujii Y, Umetani K, Shirai M (2011). Imaging of the closed-chest mouse pulmonary circulation using synchrotron radiation microangiography. J Appl Physiol (1985).

[59] Schwenke DO, Pearson JT, Umetani K, Kangawa K, Shirai M (2007). Imaging of the pulmonary circulation in the closed-chest rat using synchrotron radiation microangiography. J Appl Physiol (1985).

[60] Harish A, George T, Vm K (2008). Arteriovenous malformation after transradial percutaneous coronary intervention. Indian Heart J.

[61] Miya K, Matsushita S, Hyodo K, Tokunaga C, Sakamoto H, Mizutani T, Hiramatsu Y (2017). Renal contrast microangiography with synchrotron radiation: a novel method for visualizing structures within nephrons in vivo. Acta Radiol.

[62] Ehling J, Theek B, Gremse F, Baetke S, Mockel D, Maynard J, Ricketts SA, Grull H, Neeman M, Knuechel R (2014). Micro-CT imaging of tumor angiogenesis: quantitative measures describing micromorphology and vascularization. Am J Pathol.

[63] Ehling J, Bartneck M, Wei X, Gremse F, Fech V, Mockel D, Baeck C, Hittatiya K, Eulberg D, Luedde T (2014). CCL2-dependent infiltrating macrophages promote angiogenesis in progressive liver fibrosis. Gut.

[64] Sun D, Eirin A, Ebrahimi B, Textor SC, Lerman A, Lerman LO (2016). Early atherosclerosis aggravates renal microvascular loss and fibrosis in swine renal artery stenosis. J Am Soc Hypertens.

[65] Ehling J, Lammers T, Kiessling F (2013). Non-invasive imaging for studying anti-angiogenic therapy effects. Thromb Haemost.

[66] Advani A, Connelly KA, Yuen DA, Zhang Y, Advani SL, Trogadis J, Kabir MG, Shachar E, Kuliszewski MA, Leong-Poi H (2011). Fluorescent microangiography is a novel and widely applicable technique for delineating the renal microvasculature. PLoS ONE.

[67] Li S, Wang Y, Chen L, Wang Z, Liu G, Zuo B, Liu C, Sun D (2019). Beraprost sodium mitigates renal interstitial fibrosis through repairing renal microvessels. J Mol Med (Berl).

[68] Warner L, Glockner JF, Woollard J, Textor SC, Romero JC, Lerman LO (2011). Determinations of renal cortical and medullary oxygenation using blood oxygen level-dependent magnetic resonance imaging and selective diuretics. Invest Radiol.

[69] Pohlmann A, Arakelyan K, Seeliger E, Niendorf T (2016). Magnetic resonance imaging (MRI) analysis of ischemia/reperfusion in experimental acute renal injury. Methods Mol Biol.

[70] Pruijm M, Hofmann L, Zanchi A, Maillard M, Forni V, Muller ME, Wuerzner G, Vogt B, Stuber M, Burnier M (2013). Blockade of the renin-angiotensin system and renal tissue oxygenation as measured with BOLD-MRI in patients with type 2 diabetes. Diabetes Res Clin Pract.

[71] Li LP, Lu J, Zhou Y, Papadopoulou MV, Franklin T, Bokhary U, Solomon R, Sen A, Prasad PV (2014). Evaluation of intrarenal oxygenation in iodinated contrast-induced acute kidney injury-susceptible rats by blood oxygen level-dependent magnetic resonance imaging. Invest Radiol.

[72] Seif M, Eisenberger U, Binser T, Thoeny HC, Krauer F, Rusch A, Boesch C, Vogt B, Vermathen P (2016). Renal blood oxygenation level-dependent imaging in longitudinal follow-up of donated and remaining kidneys. Radiology.

[73] Saad A, Crane J, Glockner JF, Herrmann SM, Friedman H, Ebrahimi B, Lerman LO, Textor SC (2013). Human renovascular disease: estimating fractional tissue hypoxia to analyze blood oxygen level-dependent MR. Radiology.

[74] Chen F, Li S, Sun D (2018). Methods of blood oxygen level-dependent magnetic resonance imaging analysis for evaluating renal oxygenation. Kidney Blood Press Res.

[75] Xavier S, Vasko R, Matsumoto K, Zullo JA, Chen R, Maizel J, Chander PN, Goligorsky MS (2015). Curtailing endothelial TGF-beta signaling is sufficient to reduce endothelial-mesenchymal transition and fibrosis in CKD. J Am Soc Nephrol.

[76] Gyarmati G, Kadoya H, Moon JY, Burford JL, Ahmadi N, Gill IS, Hong YK, Der B, Peti-Peterdi J (2018). Advances in renal cell imaging. Semin Nephrol.

[77] Schiessl IM, Fremter K, Burford JL, Castrop H, Peti-Peterdi J: Long-term cell fate tracking of individual renal cells using serial intravital microscopy. *Methods Mol Biol* 2019. 10.1007/7651_2019_232 PMC6854295 31087287

[78] Schiessl IM, Grill A, Fremter K, Steppan D, Hellmuth MK, Castrop H (2018). Renal interstitial platelet-derived growth factor receptor-beta cells support proximal tubular regeneration. J Am Soc Nephrol.

[79] Iliescu R, Fernandez SR, Kelsen S, Maric C, Chade AR (2010). Role of renal microcirculation in experimental renovascular disease. Nephrol Dial Transplant.

[80] Guise E, Chade AR (2018). VEGF therapy for the kidney: emerging strategies. Am J Physiol Renal Physiol.

[81] Engel JE, Williams E, Williams ML, Bidwell GL, Chade AR (2019). Targeted VEGF (vascular endothelial growth factor) therapy induces long-term renal recovery in chronic kidney disease via macrophage polarization. Hypertension.

[82] Xu J, Wang J, Cheng Y, Li X, He M, Zhu J, Han H, Wei G, Kong H, Xie W (2018). Glucagon-like peptide-1 mediates the protective effect of the dipeptidyl peptidase iv inhibitor on renal fibrosis via reducing the phenotypic conversion of renal microvascular cells in monocrotaline-treated rats. Biomed Res Int.

[83] Singh S, Manson SR, Lee H, Kim Y, Liu T, Guo Q, Geminiani JJ, Austin PF, Chen YM (2016). Tubular overexpression of angiopoietin-1 attenuates renal fibrosis. PLoS ONE.

[84] Jialal I, Crettaz M, Hachiya HL, Kahn CR, Moses AC, Buzney SM, King GL (1985). Characterization of the receptors for insulin and the insulin-like growth factors on micro- and macrovascular tissues. Endocrinology.

[85] Liang M, Woodard LE, Liang A, Luo J, Wilson MH, Mitch WE, Cheng J (2015). Protective role of insulin-like growth factor-1 receptor in endothelial cells against unilateral ureteral obstruction-induced renal fibrosis. Am J Pathol.

[86] Ranganathan PV, Jayakumar C, Ramesh G (2013). Netrin-1-treated macrophages protect the kidney against ischemia-reperfusion injury and suppress inflammation by inducing M2 polarization. Am J Physiol Renal Physiol.

[87] Bai J, Hao J, Zhang X, Cui H, Han J, Cao N (2016). Netrin-1 attenuates the progression of renal dysfunction by blocking endothelial-to-mesenchymal transition in the 5/6 nephrectomy rat model. BMC Nephrol.

[88] Wang Z, Sun D (2018). Adipose-derived mesenchymal stem cells: a new tool for the treatment of renal fibrosis. Stem Cells Dev.

[89] Sun D, Bu L, Liu C, Yin Z, Zhou X, Li X, Xiao A (2013). Therapeutic effects of human amniotic fluid-derived stem cells on renal interstitial fibrosis in a murine model of unilateral ureteral obstruction. PLoS ONE.

[90] Wang J, Wang F, Wang Z, Li S, Chen L, Liu C, Sun D (2018). Protective effect of GDNF-engineered amniotic fluid-derived stem cells on the renal ischaemia reperfusion injury in vitro. Cell Prolif.

[91] Lu Y, Wang Z, Chen L, Wang J, Li S, Liu C, Sun D (2018). the in vitro differentiation oxf GDNF gene-engineered amniotic fluid-derived stem cells into renal tubular epithelial-like cells. Stem Cells Dev.

[92] van den Berg CW, Ritsma L, Avramut MC, Wiersma LE, van den Berg BM, Leuning DG, Lievers E, Koning M, Vanslambrouck JM, Koster AJ (2018). Renal subcapsular transplantation of PSC-derived kidney organoids induces neo-vasculogenesis and significant glomerular and tubular maturation in vivo. Stem Cell Rep.

[93] Musah S, Mammoto A, Ferrante TC, Jeanty SSF, Hirano-Kobayashi M, Mammoto T, Roberts K, Chung S, Novak R, Ingram M (2017). Mature induced-pluripotent-stem-cell-derived human podocytes reconstitute kidney glomerular-capillary-wall function on a chip. Nat Biomed Eng.

[94] Heyder C, Hansen SL, Wiesemann C (2020). Ethical aspects of translating research with human pluripotent stem cell products into clinical practice: a stakeholder approach. New Bioeth.

[95] Chen YT, Cheng BC, Ko SF, Chen CH, Tsai TH, Leu S, Chang HW, Chung SY, Chua S, Yeh KH (2013). Value and level of circulating endothelial progenitor cells, angiogenesis factors and mononuclear cell apoptosis in patients with chronic kidney disease. Clin Exp Nephrol.

[96] Chade AR, Zhu X, Lavi R, Krier JD, Pislaru S, Simari RD, Napoli C, Lerman A, Lerman LO (2009). Endothelial progenitor cells restore renal function in chronic experimental renovascular disease. Circulation.

[97] Ma YY, Sun D, Li J, Yin ZC (2010). Transplantation of endothelial progenitor cells alleviates renal interstitial fibrosis in a mouse model of unilateral ureteral obstruction. Life Sci.

[98] Tanaka T, Nangaku M (2013). Angiogenesis and hypoxia in the kidney. Nat Rev Nephrol.

[99] Li S, Zhao Y, Wang Z, Wang J, Liu C, Sun D: Transplantation of amniotic fluid-derived stem cells preconditioned with glial cell line-derived neurotrophic factor gene alleviates renal fibrosis. Cell Transplant 2018:963689718815850. 10.1177/0963689718815850 PMC6322139 30497277

[100] Saad A, Dietz AB, Herrmann SMS, Hickson LJ, Glockner JF, McKusick MA, Misra S, Bjarnason H, Armstrong AS, Gastineau DA (2017). Autologous mesenchymal stem cells increase cortical perfusion in renovascular disease. J Am Soc Nephrol.

[101] Li S, Wang Y, Wang Z, Chen L, Zuo B, Liu C, Sun D (2021). Enhanced renoprotective effect of GDNF-modified adipose-derived mesenchymal stem cells on renal interstitial fibrosis. Stem Cell Res Ther.

[102] Collett JA, Traktuev DO, Mehrotra P, Crone A, Merfeld-Clauss S, March KL, Basile DP (2017). Human adipose stromal cell therapy improves survival and reduces renal inflammation and capillary rarefaction in acute kidney injury. J Cell Mol Med.

[103] Li J, Deane JA, Campanale NV, Bertram JF, Ricardo SD (2007). The contribution of bone marrow-derived cells to the development of renal interstitial fibrosis. Stem Cells.

[104] Imafuku A, Oka M, Miyabe Y, Sekiya S, Nitta K, Shimizu T (2019). Rat mesenchymal stromal cell sheets suppress renal fibrosis via microvascular protection. Stem Cells Transl Med.

[105] Jia X, Pan J, Li X, Li N, Han Y, Feng X, Cui J (2016). Bone marrow mesenchymal stromal cells ameliorate angiogenesis and renal damage via promoting PI3k-Akt signaling pathway activation in vivo. Cytotherapy.

[106] Li S, Zhao Y, Wang Z, Wang J, Liu C, Sun D (2019). Transplantation of amniotic fluid-derived stem cells preconditioned with glial cell line-derived neurotrophic factor gene alleviates renal fibrosis. Cell Transplant.

[107] Da Sacco S, Perin L, Sedrakyan S (2018). Amniotic fluid cells: current progress and emerging challenges in renal regeneration. Pediatr Nephrol.

[108] Roemeling van Rhijn M, Reinders ME, De Klein A, Douben H, Korevaar SS, Mensah FK, Dor FJ, IJzermans JN, Betjes MG, Baan CC (2012). Mesenchymal stem cells derived from adipose tissue are not affected by renal disease. Kidney Int.

[109] Deng Y, Guo Y, Liu P, Zeng R, Ning Y, Pei G, Li Y, Chen M, Guo S, Li X (2016). Blocking protein phosphatase 2A signaling prevents endothelial-to-mesenchymal transition and renal fibrosis: a peptide-based drug therapy. Sci Rep.

[110] Smith SW, Croft AP, Morris HL, Naylor AJ, Huso DL, Isacke CM, Savage CO, Buckley CD (2015). Genetic deletion of the stromal cell marker CD248 (endosialin) protects against the development of renal fibrosis. Nephron.

[111] Mulder GM, Nijboer WN, Seelen MA, Sandovici M, Bos EM, Melenhorst WB, Trzpis M, Kloosterhuis NJ, Visser L, Henning RH (2010). Heparin binding epidermal growth factor in renal ischaemia/reperfusion injury. J Pathol.

[112] Bollee G, Flamant M, Schordan S, Fligny C, Rumpel E, Milon M, Schordan E, Sabaa N, Vandermeersch S, Galaup A (2011). Epidermal growth factor receptor promotes glomerular injury and renal failure in rapidly progressive crescentic glomerulonephritis. Nat Med.

[113] Miyazawa T, Zeng F, Wang S, Fan X, Cheng H, Yang H, Bian A, Fogo AB, Harris RC (2013). Low nitric oxide bioavailability upregulates renal heparin binding EGF-like growth factor expression. Kidney Int.

[114] Zeng F, Kloepfer LA, Finney C, Diedrich A, Harris RC (2016). Specific endothelial heparin-binding EGF-like growth factor deletion ameliorates renal injury induced by chronic angiotensin II infusion. Am J Physiol Renal Physiol.

[115] Sun D, Ma Y, Han H, Yin Z, Liu C, Feng J, Zhou X, Li X, Xiao A, Yu R (2012). Thrombospondin-1 short hairpin RNA suppresses tubulointerstitial fibrosis in the kidney of ureteral obstruction by ameliorating peritubular capillary injury. Kidney Blood Press Res.

[116] McGregor B, Colon S, Mutin M, Chignier E, Zech P, McGregor J (1994). Thrombospondin in human glomerulopathies. A marker of inflammation and early fibrosis. Am J Pathol.

[117] Liu B, Qiang L, Wang GD, Duan Q, Liu J (2019). LncRNA MALAT1 facilities high glucose induced endothelial to mesenchymal transition and fibrosis via targeting miR-145/ZEB2 axis. Eur Rev Med Pharmacol Sci.

